# Magnesium Intake, Sociodemographic Determinants, and Memory Performance in Older Adults: A Sex-Stratified Analysis in NHANES 2011–2014

**DOI:** 10.3390/biology15211906

**Published:** 2026-10-22

**Authors:** Huma Shahzad, Mohammed S. Razzaque, Juan Carlos Lopez-Alvarenga

**Affiliations:** 1 Department of Medical Education, School of Medicine, University of Texas Rio Grande Valley, Edinburg, TX 78539, USA; huma.shahzad@utrgv.edu (H.S.); mohammed.razzaque@utrgv.edu (M.S.R.); 2 Division of Population Health and Biostatistics, University of Texas Rio Grande Valley, Edinburg, TX 78539, USA

**Keywords:** aged, cognition, magnesium, memory, nutrition process, structural equation modeling

## Abstract

Background: Magnesium intake (MgI) has been associated with cognitive health in older adults, but the influence of sex, socioeconomic factors, and overall dietary intake remains unclear. Methods: We analyzed adults aged ≥ 60 years from the National Health and Nutrition Examination Survey (NHANES) 2011–2014. Of 3079 participants eligible before missing data exclusions, 2397 (1153 men and 1244 women) then had complete information for the final structural equation model (SEM). Total magnesium intake included dietary and supplemental sources assessed using two 24-h dietary recalls. Memory performance was derived using principal-component factor analysis of CERAD word-recall and animal-fluency assessments. Survey-weighted, sex-specific SEMs evaluated associations among magnesium intake, memory performance, age, education, household income, and total energy intake. Results: Participants had a mean age of 70 ± 6.8 years, 52% were female, and mean BMI was 29 ± 6.4 kg/m^2^. Without energy adjustment, total magnesium intake showed a positive but nonsignificant association with memory in men (β = 0.043, *p* = 0.387) and women (β = 0.080, *p* = 0.105). After energy adjustment, the associations were attenuated in men (β = 0.030, *p* = 0.486) and women (β = 0.040, *p* = 0.532). Energy intake was strongly associated with magnesium intake but not with memory. Education was positively associated with memory, whereas age was negatively associated in both sexes. Conclusion: Total magnesium intake was not independently associated with memory performance after accounting for energy intake and sociodemographic factors. Education and age remained the most consistent predictors of memory. These findings highlight the importance of considering overall dietary intake when examining nutrient–cognition relationships.

## Simple Summary

Magnesium is an essential nutrient found in foods such as leafy green vegetables, nuts, seeds, and whole grains, and is also commonly taken in dietary supplements. We studied whether total magnesium intake from food and supplements was related to memory in 2397 U.S. adults aged 60 years and older who participated in a national health and nutrition survey. Magnesium intake showed a small positive relationship with memory before considering total energy intake, particularly among women, but the relationship was not statistically significant. People who consumed more magnesium also tended to consume more calories overall. After taking energy intake into account, there was no clear relationship between magnesium intake and memory in either men or women. In contrast, education and age remained consistently related to memory performance. These findings do not rule out a role for magnesium in brain health, but they suggest that its relationship with memory is difficult to separate from overall diet and social factors. Because participants were studied at one point in time, we cannot determine whether magnesium intake affects memory. Long-term and intervention studies are needed to better understand the role of magnesium in healthy brain aging.

## 1. Introduction

Magnesium is a vital mineral that plays a crucial role in controlling several key biological functions, including blood glucose regulation, muscle and nerve function, blood pressure regulation, and protein synthesis. It is found in the human body in at least three different pools: skeletal muscles, intracellular, and extracellular [1,2]. Magnesium is required for several metabolic reactions, and the long-term effect of this low intake has not been adequately reported [3]. Nevertheless, Magnesium deficiency is increasingly common among the elderly and is associated with a range of symptoms and health issues that can significantly impact quality of life. Approximately 60% of adults in the United States do not consume the dietary reference intake for magnesium, and magnesium deficiency may be even more common because of increased incidence of metabolic syndrome and type 2 diabetes mellitus, both of which increase magnesium excretion [3]. As people age, several factors contribute to magnesium deficiency, including decreased dietary intake, impaired intestinal absorption, and reduced renal function, which can result in lower blood magnesium levels [4].

Magnesium acts as a cofactor for many enzymes involved in neurotransmitter synthesis and neural signaling; its deficiency disrupts cognition [4]. Both low and high magnesium levels have been associated with an increased risk of memory decline, including dementia [4,5]. Magnesium participates in controlling synapse plasticity, stabilizing the NMDA receptor, reducing neural noise, and promoting synaptic plasticity [6,7]. Animal experiments have shown that magnesium L-threonate, a customized supplement, enhances hippocampal synaptic density, thereby improving learning, working memory, and long-term memory [8]. Human studies also support cognitive benefits [9]. Dietary sources of magnesium from leafy greens, nuts, seeds, and whole grains are associated with larger brain volume and fewer white matter lesions [10].

Recent evidence suggests that magnesium metabolism differs between men and women. Men have larger body stores due to their muscular mass, and women are more sensitive to hormonal status [11]. Estrogens increase intestinal absorption and renal retention of magnesium, and in women, higher dietary MgI is associated with better brain health [10]. Clinical patterns also differ between sexes; for instance, magnesium deficiency in women is linked to osteoporosis, preeclampsia, and postmenopausal bone loss [11], whereas in men, it is more closely associated with insulin resistance and cardiovascular risk, but this association still needs to be studied [12]. However, few clinical trials are designed to analyze the effects of magnesium supplementation by sex, and the specific mechanisms underlying these effects and their relationship with other diseases, such as diabetes or chronic kidney disease, are incompletely understood [13].

Magnesium plays a crucial role in cognitive function and memory, particularly among the elderly, and its deficiency can have significant consequences. With the aging population, the risk of memory loss and dementia becomes widespread, impacting individuals, caregivers, and health systems. Records show that ~62.7 million Americans are aged 65+ (~18.6% of the population). The numbers are likely to exceed 70 million (≈~20%) by 2030 [14], and with that, the ~7 million dementia cases in 2020 are expected to grow to over 9 million by 2030 [15]. Currently, ~30–33% of older Americans experience noticeable memory or cognitive issues [16]. However, using modifiable factors, such as addressing magnesium deficiency through diet and supplementation, could potentially help prevent memory decline and substantially reduce the likelihood and delay the progression of memory decline in elderly individuals.

Our primary objective was to assess the impact of magnesium status on cognitive health in adults older than 60 years. We addressed the gap in knowledge on sex metabolism and its potential role in preventing cognitive decline, considering biological and social factors.

## 2. Materials and Methods

The National Health and Nutrition Examination Survey (NHANES) is a cross-sectional survey designed to monitor the health and nutritional status of a nationally representative sample of the noninstitutionalized civilian U.S. population. NHANES uses a complex, multistage probability sampling design. Analyses incorporated the masked variance pseudo-strata (variable named SDMVSTRA), pseudo-primary sampling units (variable named SDMVPSU), and dietary sampling weights. Because the final magnesium and energy exposures were calculated from the mean of two 24-h dietary recalls, participants were required to have valid dietary data from both Day 1 and Day 2. Accordingly, the two-day dietary recall weight (WTDR2D) was used. The 2-year dietary weights from the 2011–2012 and 2013–2014 cycles were divided by two to generate the appropriate 4-year analytic weight. Survey analyses were conducted for the subpopulation of adults aged ≥ 60 years.

**Figure 1:**
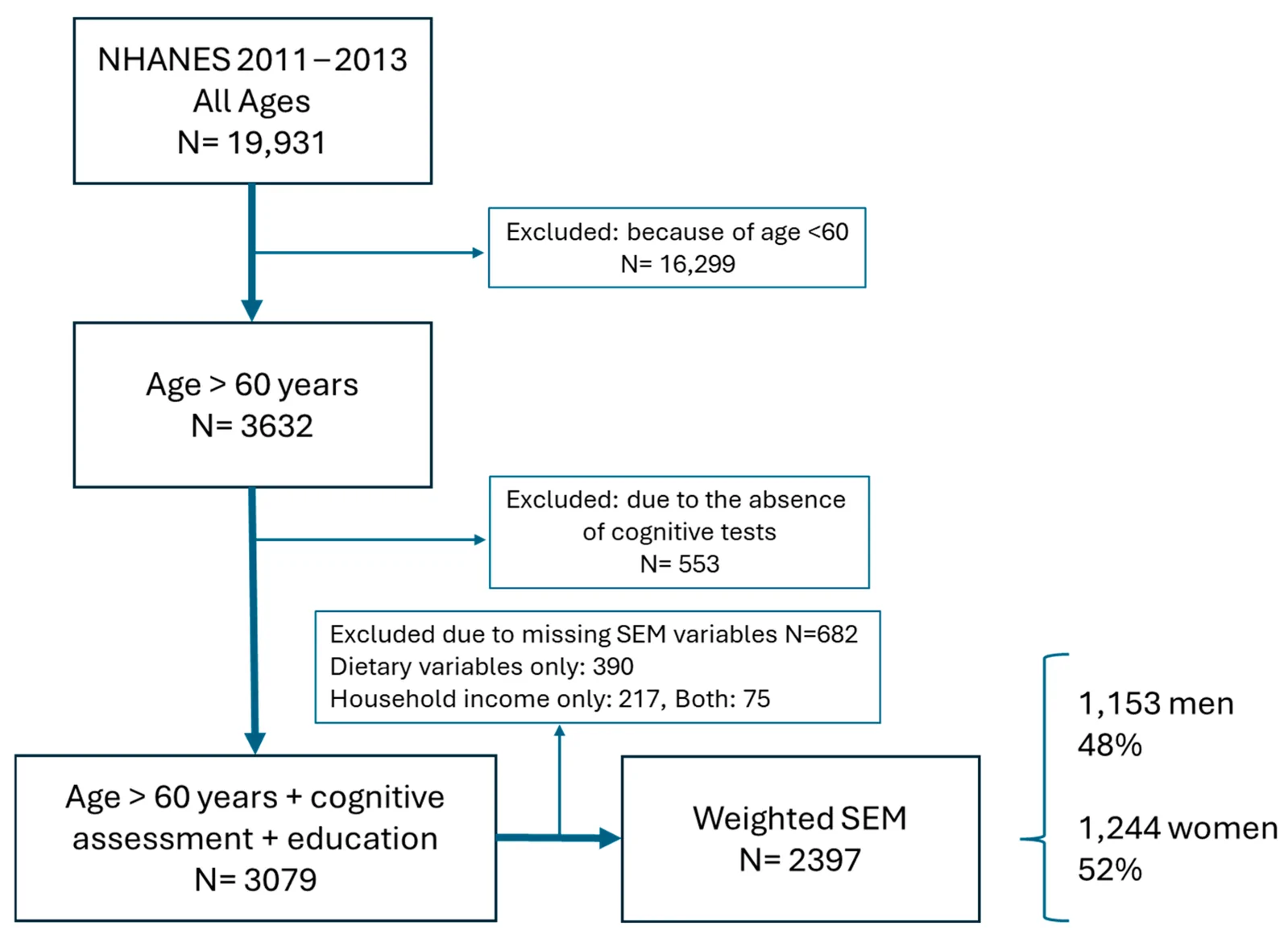
Participant selection flowchart for the NHANES 2011–2014 magnesium intake and memory study. The final survey-weighted, sex-specific SEM included 2397 participants (1153 men and 1244 women). Numbers represent unweighted participant counts.

The study sample was 3083 participants with cognitive data required for the analysis. The final survey-weighted SEM required valid Day 1 and Day 2 dietary recalls and complete magnesium, energy intake, and valid covariates (age, education, and household income). These requirements reduced the analytic sample to 2397 participants (1153 men and 1244 women, Figure 1).

Cognitive performance was assessed using data from the NHANES Cognitive Functioning Questionnaire (CFQ). The present analysis included the Consortium to Establish a Registry for Alzheimer’s Disease (CERAD) Word Learning test and the Animal Fluency test. The CERAD assessment included three immediate word-recall trials and a delayed-recall trial. For each trial, NHANES recorded the number of correctly recalled words and the number of intrusion words. Animal Fluency was assessed by asking participants to name as many animals as possible within one minute, with the total number of correct responses recorded. The assessments were administered by trained interviewers using standardized NHANES procedures in the Mobile Examination Center.

To reduce redundancy among correlated cognitive measures and avoid analyzing overlapping test scores as independent outcomes, we used principal-component factor analysis (PCF) to summarize their shared information into composite dimensions. PCF with orthogonal Varimax rotation was performed using nine variables: the three CERAD immediate-recall scores, CERAD delayed recall, the four corresponding intrusion-word counts, and Animal Fluency. Sampling adequacy was good (overall KMO = 0.834; individual KMO values = 0.76–0.94). Two factors accounted for 61.2% of the total variance. Factor 1 explained 39% and showed strong loadings for the three immediate recall trials (0.80–0.87), delayed recall (0.84), and Animal Fluency (0.58), whereas the intrusion-word measures loaded primarily on the second factor. Based on this structure, Factor 1 was interpreted as memory performance (F1mp) and was used as the cognitive outcome in subsequent analyses; the second factor was not included in the present analysis.

**Ingestion of Magnesium**. The magnesium intake (MgI) was calculated using detailed information from questionnaires. The What We Eat in America (WWEIA) [17] is a dietary interview used to estimate the types and amounts of foods and beverages consumed during the 24 h preceding the interview. All participants had two 24-h dietary recall interviews. The first was collected in person, and the second was collected by phone, 3 to 10 days after the first. Detailed procedures were found in the Data Documentation, Codebook, and the frequencies webpage [18]. Briefly, the WWEIA utilized dietary data collected from an application to analyze food and beverage items, grouping them by similar categories based on usage and nutrient content [19]. For the SEM weighted analysis, we required valid data from both recalls.

The 2011–2014 NHANES cycles were selected because they provide the cognitive assessments required for the present analysis, including CERAD word learning and recall and Animal Fluency, in adults aged 60 years and older. In addition to the dietary recalls, NHANES collected information on dietary supplements consumed during the same 24-h periods. Magnesium from supplements was obtained from the Day 1 and Day 2 total dietary supplement files, separately from magnesium derived from foods and beverages. For each recall day, supplemental magnesium intake (mg/day) was added to dietary magnesium intake from the corresponding dietary recall. Total magnesium intake was therefore calculated as the sum of magnesium from foods and beverages and magnesium from dietary supplements for each day.

For participants with valid data from both recall days, mean dietary magnesium, mean supplemental magnesium, and mean total magnesium intake were calculated across Days 1 and 2. The primary magnesium exposure used in the final analyses was mean total magnesium intake from dietary and supplemental sources. Because nutrient intake is related to the overall amount of food consumed, mean energy intake was also calculated from the two dietary recalls and included in the final structural equation model. The two-day dietary sampling weights were used for analyses based on the two-day dietary intake data.

Variables with heavy tails (most had right-tail skewness) were transformed to natural logarithms; however, extreme kurtosis, due to its nature, was transformed using the inverse normalization method with the Van der Waerden approach (not used in the structural equation modeling).

**Magnesium by demographic variables**. Differences in magnesium intake by survey cycle and sex were initially examined using mean comparisons, with Hedges’ g calculated to describe the magnitude of between-group differences. Associations between magnesium intake and sociodemographic characteristics, including education, marital status, and household income, were further examined using ordinary least squares regression. Post-estimation pairwise comparisons were performed with bootstrapped standard errors for models using untransformed magnesium intake.

**Structural Equation Modeling (SEM)**. SEM was used to examine associations among sociodemographic characteristics, magnesium intake (MgI), adiposity, blood pressure, and memory performance (F1mp). Initial exploratory models were estimated without survey weights to evaluate alternative model structures. The analyses began with a simple model examining the associations of body mass index (BMI) and daily magnesium intake with memory performance. More complex models were subsequently evaluated by incorporating adiposity as a latent construct represented by observed anthropometric indicators, including BMI, waist circumference, sagittal abdominal diameter, and arm circumference. Blood pressure and magnesium intake were specified as endogenous variables. Variables were standardized to facilitate interpretation of the estimated coefficients. Model performance was examined using the root mean square error of approximation (RMSEA), standardized root mean square residual (SRMR), and Bayesian information criterion (BIC). The addition of adiposity and blood pressure did not provide sufficient improvement in model fit or explanatory performance to justify retaining these components. Consequently, a more parsimonious model was selected.

Subsequent models incorporated education, household income, and age as observed exogenous variables associated with both magnesium intake and memory performance. Total energy intake was subsequently included to account for differences in overall dietary consumption. In the final model, magnesium intake and memory performance were specified as observed endogenous variables, with magnesium intake occupying an intermediate position in the structural model. The relationships were interpreted as statistical associations rather than causal or mediating effects.

The final model was estimated using survey-weighted multigroup SEM, with separate coefficients for men and women, accounting for the complex NHANES sampling design. The analytical sample included 2397 participants (1153 men and 1244 women) with complete information for the variables included in the model. Conventional likelihood-based fit indices, including the chi-square goodness-of-fit statistic, comparative fit index (CFI), RMSEA, and BIC, were considered during exploratory model development but were not used to evaluate the final survey-adjusted model. Instead, model evaluation was based on the available SRMR, overall coefficient of determination, and equation-level R^2^ for the endogenous variables. Sex-stratified coefficients were reported descriptively, without interpreting differences between men and women as statistically significant in the absence of formal between-group comparisons. 

All analyses were performed using Stata MP version 19 (StataCorp, College Station, TX, USA).

## 3. Results

### 3.1. Main Sample Characteristics

The process of sample selection is described in Figure 1. The final cognitive sample was N = 3083, including 48% men and 52% women, with a mean age of 70 years (SD = 6.8). The final sample for complete-case SEM was N = 2397.

A description of the population’s characteristics is presented in Table 1. The average body mass index (BMI) was 29.3 (95% CI: 28.8, 29.8) kg/m^2^. Among adults aged ≥ 60 years the survey-weighted mean dietary magnesium intake was 294.2 (95% CI: 283.3, 305.1) mg/day, while mean supplemental magnesium intake was 48.3 (95% CI: 40.6, 56.0) mg/day. After combining dietary and supplemental sources, the mean total magnesium intake was 342.5 (95% CI: 327.4, 357.6) mg/day.

### 3.2. Magnesium Levels by Groups

The Mg levels by year did not show a difference between years [Difference MgI 0.009 (95% CI −0.041, 0.023) natural logarithm of mg/day (*p* = 0.56)] with a Hedge’s g of 0.02 (95% CI −0.05, 0.10). The difference between sexes was statistically significant [Difference in MgI 0.169 (95% CI 0.14, 0.20)], favoring men with a moderate effect size, Hedge’s g of 0.40 (95% CI 0.32, 0.47).

**Figure 2:**
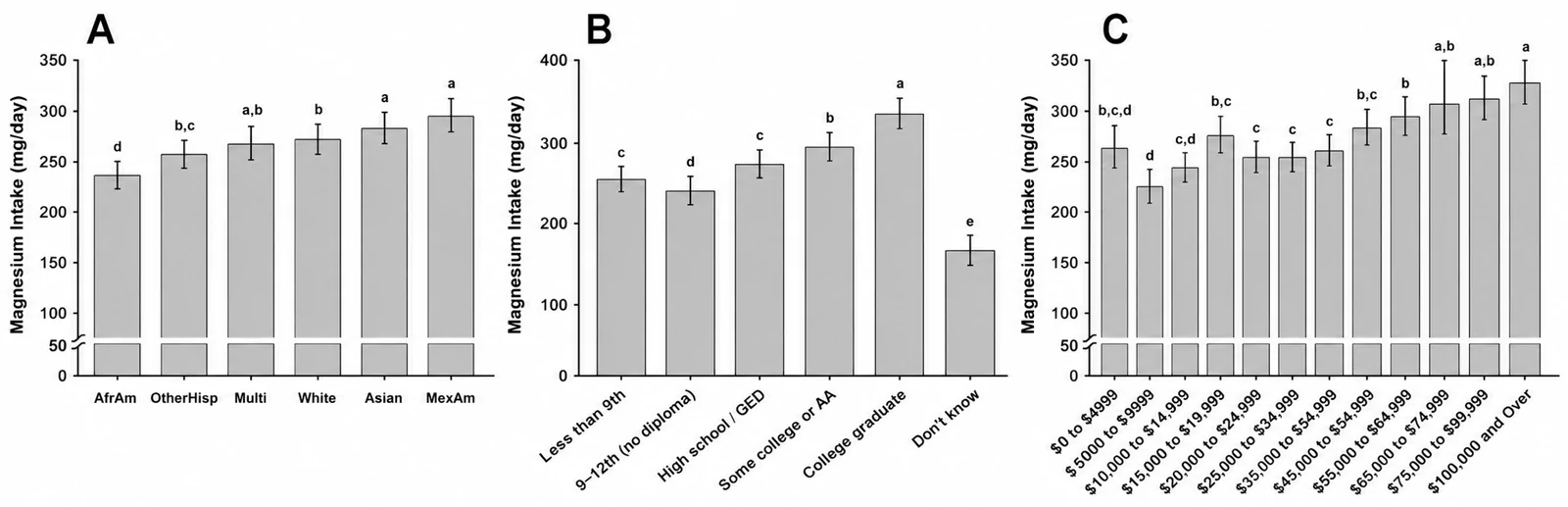
Magnesium intake by sociodemographic characteristics. The bar graphs show magnesium intake (mg/day) by (**A**) race/ethnicity, (**B**) educational attainment, and (**C**) bootstrapping standard errors. Superscript letters denote statistically significant pairwise differences based on post hoc comparisons (Bonferroni-adjusted; *p* < 0.05). Groups sharing a letter are not significantly different. AfrAm = African American; MexAm = Mexican American; OtherHisp = Other Hispanic; Multi = Multiracial; AA = Associate’s Degree; GED = General Educational Development.

The mean MgI varied significantly across different racial and ethnic groups, educational levels, and income levels (Figure 2). Among racial/ethnic groups, Mexican Americans and Asians had the highest MgI, while African Americans had the lowest (*p* < 0.001). Educational attainment was also associated with intake (*p* < 0.001). Participants with a college degree had the highest MgI, while individuals with less than 9th-grade education or those who did not report on education had the lowest values. Notably, the “Don’t know” group was significantly lower than all others. The household income showed a clear positive gradient in MgI (*p* < 0.001).

### 3.3. Principal-Component Factor Analysis (PCF) with Varimax Rotation

The PCF with Varimax rotation was applied to nine cognitive performance variables: three immediate recalls, one delayed recall, three intrusion errors, one delayed intrusion, and an animal fluency task. The overall Kaiser-Meyer-Olkin (KMO) was 0.83, indicating suitable intercorrelations. Two factors were retained with Varimax rotation, explaining 61.2% of the total variance. Factor 1 reflects a “Memory Performance” construct; meanwhile, Factor 2 represents a “Verbal Intrusion/Interference” construct (Supplemental Table S1). The standardized residuals and the anti-image matrix supported this two-factor structure (Supplementary Figure S1). For the purpose of the current analysis, we focused on Factor 1.

### 3.4. Structural Equation Models

**Figure 3:**
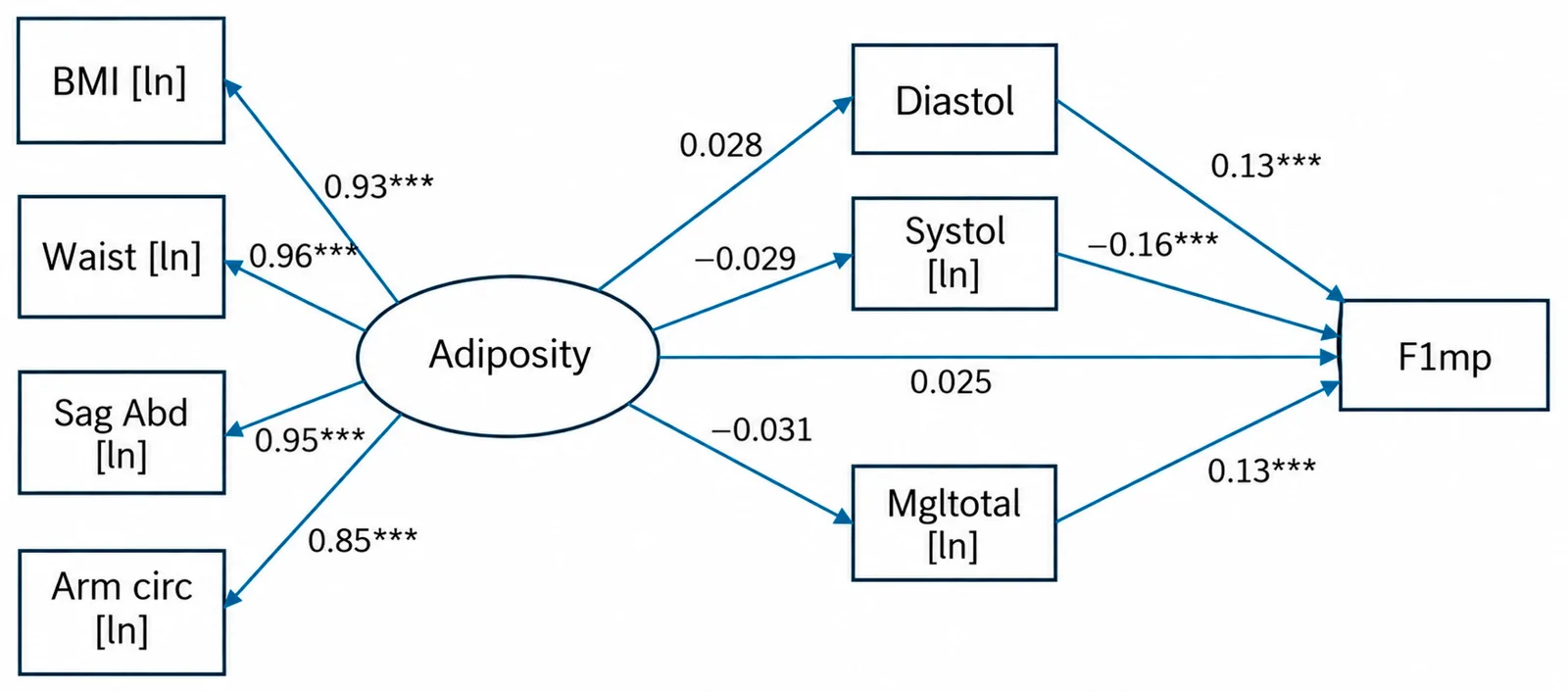
Structural equation model evaluating adiposity, magnesium intake, blood pressure, and memory performance (F1mp). The model showed substantial explained variance in the measurement component (R^2^ = 0.968), although overall model fit was poor (RMSEA = 0.175, 90% CI: 0.164–0.187; SRMR = 0.066; BIC = 11,536). Adiposity was not significantly associated with memory performance, blood pressure, or magnesium intake. Significant direct associations with memory performance were observed for systolic blood pressure (β = −0.16), diastolic blood pressure (β = 0.13), and magnesium intake (β = 0.13). Replacing BMI with waist circumference did not materially improve the model. BMI [ln], natural logarithm of body mass index; Sag Abd, sagittal abdominal diameter; Arm circ, arm circumference; Diastol/Systol, diastolic/systolic blood pressure; MgI, magnesium intake; F1mp, memory performance factor. * *p* < 0.10, ** *p* < 0.05, *** *p* < 0.001.

An initial SEM evaluated the relationships among adiposity, magnesium intake (MgI), blood pressure, and memory performance (F1mp). Although the model showed substantial explained variance in its measurement component (R^2^ = 0.97), overall model fit was poor (RMSEA = 0.175, 90% CI: 0.164–0.187; SRMR = 0.066; BIC = 11,536; Figure 3). In the structural component, systolic blood pressure (β = −0.16, *p* < 0.001), diastolic blood pressure (β = 0.13, *p* < 0.001), and MgI (β = 0.13, *p* < 0.001) were associated with memory performance. Adiposity was not associated with memory performance (β = 0.02, *p* = 0.375), systolic blood pressure (β = −0.03, *p* = 0.331), diastolic blood pressure (β = 0.03, *p* = 0.335), or MgI (β = −0.03, *p* = 0.291). Replacing BMI with waist circumference did not materially improve the model. Therefore, adiposity and blood pressure were not retained in the final parsimonious model.

**Figure 4:**
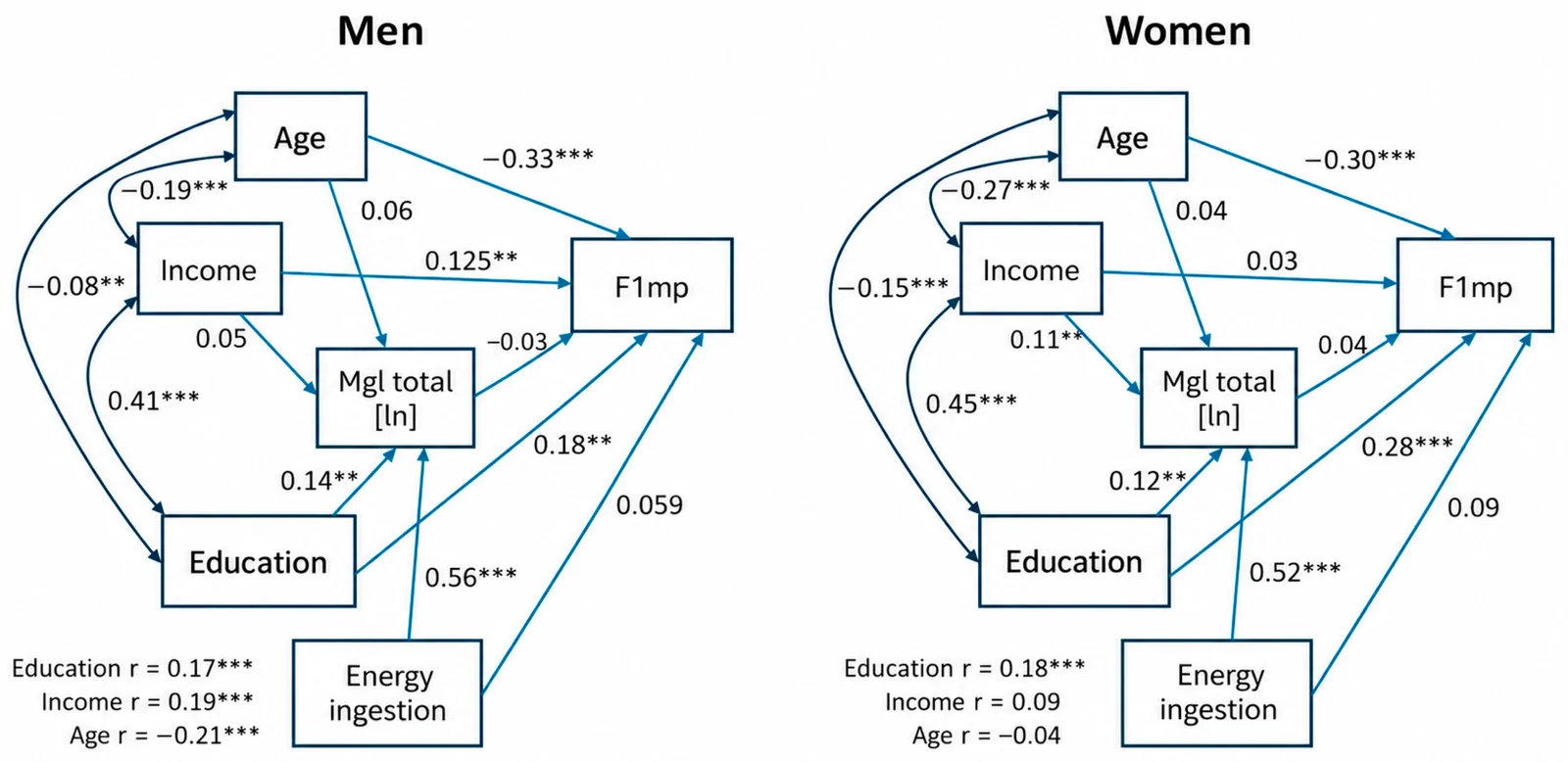
Sex-stratified structural equation models of total magnesium intake and memory performance (F1mp) adjusted for energy intake. Total magnesium intake was log-transformed, and energy intake was expressed per 100 kcal/day. Paths values represent standardized coefficients (β). Curved double-headed arrows represent covariances among sociodemographic variables. To simplify the figure, correlations (r) between energy intake and the sociodemographic variables are reported beside the energy intake box rather than displayed as double-headed arrows. Analyses considered weights for the NHANES complex survey design. * *p* < 0.10, ** *p* < 0.05, *** *p* < 0.001.

The final survey-weighted SEM incorporated total magnesium intake from dietary and supplemental sources and was adjusted for total energy intake, age, education, and household income. This survey-weighted model included 2397 participants (1153 men and 1244 women) and accounted for the NHANES complex sampling design using 29 strata and 61 primary sampling units (Figure 4). The SRMR was <0.001 and the overall coefficient of determination was 0.496. At the equation level, the model explained 23.1% of the variance in memory performance and 39.2% of the variance in magnesium intake among men, and 24.1% and 33.4%, respectively, among women. Total energy intake was strongly associated with total magnesium intake in both men (β = 0.583, 95% CI: 0.513, 0.653; *p* < 0.001) and women (β = 0.516, 95% CI: 0.454, 0.579; *p* < 0.001). After accounting for energy intake and sociodemographic factors, total magnesium intake was not independently associated with memory performance in men (β = 0.030, 95% CI: −0.057, 0.117; *p* = 0.173) or women (β = 0.039, 95% CI: −0.089, 0.168, *p* = 0.532). Education remained positively associated with memory performance in both men (β = 0.181, *p* = 0.003) and women (β = 0.279, *p* < 0.001), whereas age was negatively associated with memory performance in men (β = −0.324, *p* < 0.001) and women (β = −0.303, *p* < 0.001).

### 3.5. Sensitivity Analyses

To examine the influence of energy adjustment, the final model was repeated without total energy intake while retaining total magnesium intake from dietary and supplemental sources (Supplementary Figure S2). In this model, the association between total magnesium intake and memory performance remained positive but was not statistically significant in either men (β = 0.043, 95% CI: −0.056–0.142, *p* = 0.387) or women (β = 0.080, 95% CI: −0.018–0.178, *p* = 0.105). Adjustment for energy intake further attenuated these associations, particularly among men. Across both models, education and age remained the predominant predictors of memory performance. The standardized coefficients from the models with and without energy adjustment are compared in Supplementary Table S2.

Sensitivity analysis adjusting for race/ethnicity. To evaluate potential residual confounding, we repeated the survey-weighted, sex-stratified SEM with race/ethnicity included as an additional covariate. The association between total magnesium intake and memory performance remained nonsignificant in men (β = 0.032, 95% CI: −0.053 to 0.117; *p* = 0.446) and women (β = 0.038, 95% CI: −0.092 to 0.167; *p* = 0.555). These estimates were similar to those obtained in the primary model without race/ethnicity adjustment (β = 0.030 in men and β = 0.040 in women), suggesting that the principal findings were not materially influenced by the additional adjustment.

### 3.6. Comparison of Included and Excluded Participants

To assess potential selection bias in the complete-case analysis, we compared participants included in the final SEM (n = 2397) with those excluded because of missing information (n = 682). Excluded participants had significantly lower memory performance scores than included participants (−0.261 ± 1.074 vs. 0.076 ± 0.964; mean difference = −0.337, 95% CI: −0.421 to −0.252; *p* < 0.001). Educational attainment also differed significantly between groups (χ^2^(4) = 64.88; *p* < 0.001), with excluded participants more likely to have less than a high school education. In contrast, age (70.0 ± 0.266 vs. 69.7 ± 0.14; *p* = 0.152) and sex distribution did not differ significantly (χ^2^(1) = 0.55; *p* = 0.459). These findings suggest that complete-case selection may disproportionately exclude participants with poorer memory performance and lower educational attainment, potentially limiting the generalizability of the SEM findings.

## 4. Discussion

In our study, total magnesium intake, including both dietary and supplemental sources, was not independently associated with memory performance in older men or women. Before adjustment for energy intake, the association was positive but did not reach statistical significance in either men (β = 0.043, *p* = 0.387) or women (β = 0.080, *p* = 0.105). After adjustment for total energy intake, these associations were further attenuated (β = 0.030 in men and β = 0.040 in women). In contrast, education remained positively associated with memory performance, while age was consistently negatively associated with memory in both sexes. These findings suggest that sociodemographic factors, particularly education and age, were more consistent predictors of memory performance than total magnesium intake in this population.

The attenuation after energy adjustment is important for interpreting the magnesium findings. Previous studies suggest men have greater total body magnesium stores, but women have a hormonal influence on magnesium homeostasis [11]. Total magnesium intake was strongly associated with energy intake in both men and women, which is expected because dietary magnesium intake is partly determined by the overall amount of food consumed. Although supplemental magnesium is not directly linked to caloric intake, dietary sources contributed substantially to total magnesium exposure. Energy adjustment therefore addresses a more specific question: whether total magnesium intake is associated with memory performance beyond differences related to overall energy consumption. The absence of a statistically significant association after adjustment does not demonstrate that magnesium is biologically unrelated to cognition. Rather, an independent magnesium-specific association with memory could not be demonstrated in this population. Moreover, because the association was already nonsignificant in the model without energy adjustment, the findings should not be interpreted as showing that energy adjustment alone eliminated a magnesium effect. Given the cross-sectional design and the close relationship between nutrient and energy intake, we cannot distinguish the potential effects of magnesium from those of broader dietary patterns and other correlated nutritional factors.

In the initial models, blood pressure, particularly systolic blood pressure, showed modest associations with memory performance. However, these relationships were not retained in the final parsimonious model after sociodemographic factors were considered. Likewise, adiposity, assessed using BMI and waist circumference, did not provide meaningful additional explanatory value for magnesium intake or memory performance. In contrast, education and age showed consistent associations with memory across the sex-stratified models. Household income showed weaker and less consistent associations. The strong relationship between education and memory is compatible with the concept of cognitive reserve, whereby educational attainment may contribute to resilience against age-related cognitive decline. These results emphasize the importance of considering social and demographic factors when evaluating nutritional associations with cognitive performance in older adults.

Previous studies have reported positive associations between magnesium intake and cognitive performance. An analysis of NHANES data by Tao et al. found that higher dietary magnesium intake was associated with better cognitive performance, including immediate and delayed recall [20]. Despite the common data source, several methodological differences may explain the apparent discrepancy. Tao et al. estimated total magnesium intake using a single dietary recall day and supplement information, categorized exposure into quartiles, and evaluated a global cognitive z-score through survey-weighted linear regression. In contrast, we averaged total magnesium intake across two days, modeled its natural logarithm continuously, and examined a memory-specific factor score using sex-stratified, survey-weighted structural equation modeling. The studies also differed in covariate selection, although both adjusted for energy intake. Notably, Tao et al. found no statistically significant associations with CERAD immediate or delayed recall after full adjustment, despite their positive finding for global cognition. This suggests that the relationship between magnesium intake and cognitive performance may differ across cognitive domains and analytical specifications. Accordingly, our results do not necessarily contradict Tao et al., but rather highlight the importance of outcome selection and modeling decisions when interpreting magnesium–cognition associations.

Studies using biochemical measures of magnesium status have also reported associations with cognitive outcomes. Among Qatari adults, lower serum magnesium concentrations were associated with slower cognitive reaction time, suggesting a potential relationship between magnesium status and cognitive performance [21]. However, these findings should be interpreted cautiously because circulating magnesium concentrations and dietary magnesium intake represent different measures of exposure.

Our findings provide a more cautious interpretation. Although the direction of the association between total magnesium intake and memory was positive before energy adjustment, particularly among women, the confidence intervals included the null. The associations were further attenuated after energy intake was included in the model. Differences in the definition of magnesium exposure, inclusion of dietary supplements, dietary assessment, energy adjustment, cognitive outcomes, study populations, and covariate selection may contribute to differences across studies. Our findings therefore highlight the importance of considering both supplemental intake and overall energy consumption when evaluating associations between individual nutrients and cognitive outcomes.

Magnesium nevertheless has biological functions that provide a plausible basis for its potential involvement in cognitive health. Magnesium participates in cellular energy metabolism, mitochondrial function, neuronal signaling, and mechanisms involved in synaptic plasticity [22]. Research indicates that magnesium plays a vital role in neuroprotection, and deficiency is linked to neurodegenerative processes such as amyloid-beta (Aβ) accumulation, which is associated with Alzheimer’s disease and other forms of dementia [22]. However, evidence linking magnesium status to neurodegenerative disorders remains primarily observational. A systematic review and meta-analysis reported lower serum and plasma magnesium concentrations among patients with Alzheimer’s disease compared with healthy controls [23]. Similarly, a pilot study involving older adults with vascular cognitive impairment found that lower intra-erythrocyte magnesium concentrations, but not plasma magnesium concentrations, were associated with cognitive impairment and its severity [24]. These findings suggest that different measures of magnesium status may capture distinct aspects of magnesium homeostasis, although they support continued investigation of magnesium in cognitive aging. However, they should not be interpreted as evidence that increasing magnesium intake will improve memory based on the present study. Our cross-sectional findings did not demonstrate an independent association between total magnesium intake and memory after accounting for energy intake and sociodemographic factors. Longitudinal studies and randomized clinical trials are needed to determine whether magnesium intake or status contributes independently to cognitive health.

**Used methods**. Our analyses included supplemental magnesium. Mean dietary magnesium intake, supplements, and their aggregation provided a more complete estimate of magnesium exposure and may partly explain differences between our findings and studies based exclusively on dietary magnesium.

With the PCF with Varimax rotation, the anti-image diagonals for the second and third immediate recalls were lower than ideal; their factor loadings were greater than 0.83, indicating substantial shared variance with the primary memory construct. The reduced anti-image values may stem from redundancy among the three immediate recall trials or practice effects, but removing these would risk underrepresenting the sequential memory process. Furthermore, the structure matrix confirms the coherence of these variables with the recall factor. Therefore, they were retained to maintain theoretical completeness and statistical integrity of the memory domain.

The survey-weighted, sex-stratified SEM was overidentified (df = 18). The standardized root mean squared residual (SRMR) was <0.001, and the overall coefficient of determination was 0.496. Given the restrictions on conventional global fit statistics under survey-weighted estimation, the SRMR was interpreted cautiously rather than as definitive evidence of model adequacy.

**Strengths**. This study used NHANES, a nationally representative survey of the U.S. population, and incorporated its complex sampling design into the analyses. Magnesium intake was estimated from two 24-h dietary recalls and expanded to include magnesium from dietary supplements reported during the corresponding 24-h periods, providing a more comprehensive measure of total magnesium exposure. Mean energy intake was derived from the same two dietary recalls, and the corresponding two-day dietary sampling weights were applied. The use of established cognitive assessments, including CERAD word recall and animal fluency, allowed us to characterize memory performance using a data-driven factor approach. Structural equation modeling further allowed us to examine the relationships among nutritional, sociodemographic, and cognitive factors simultaneously, while sex-stratified analyses identified differences in the magnitude and pattern of these associations. Finally, comparing models with and without energy adjustment provided an assessment of whether the magnesium–memory relationship was independent of overall energy intake.

**Limitations**. Several limitations should be considered. First, the cross-sectional design precludes causal inference or determination of the temporal relationship between magnesium intake and memory performance. Second, the complete-case analysis excluded participants with missing information and may therefore be susceptible to selection bias. Dietary intake was based on self-reported 24-h recalls and remains subject to recall and measurement error; two recall days provide a better estimate than a single day but may still not adequately represent an individual’s usual long-term intake. Although the exposure incorporated magnesium from both dietary and supplemental sources, it represents intake during the two 24-h assessment periods rather than long-term magnesium status and does not provide a biomarker of magnesium stores.

Complete-case analysis excluded 682 of 3079 eligible participants (22.2%). Comparisons between included and excluded participants showed significantly lower memory performance and educational attainment among those excluded, whereas age and sex distributions were similar. These differences suggest that missingness was associated with characteristics relevant to cognitive performance and may have introduced selection bias. Therefore, the estimated magnesium–memory associations may not fully represent the eligible population, and the null findings should be interpreted cautiously. The direction and magnitude of any resulting bias cannot be determined from these comparisons alone.

Total magnesium intake was also strongly associated with energy intake, largely reflecting the contribution of food intake to magnesium exposure. This correlation makes it difficult to isolate a magnesium-specific association from the broader dietary patterns in which magnesium is consumed. Although energy intake and several sociodemographic factors were considered, residual confounding from physical activity, medication use, comorbidities, dietary quality, and other nutrients remains possible. Finally, the analysis focused primarily on memory-related performance and did not comprehensively evaluate other cognitive domains.

## 5. Conclusions

In adults aged 60 years and older, total magnesium intake from dietary and supplemental sources was not independently associated with memory performance. The association was modest and positive before adjustment for total energy intake, particularly among women, but did not reach statistical significance and was further attenuated after energy adjustment. Because total magnesium intake was strongly related to overall energy consumption, these findings highlight the difficulty of separating a magnesium-specific association from the broader dietary context in which magnesium is consumed. However, they do not exclude a potential biological role of magnesium in cognitive health. Age and education remained the most consistent predictors of memory performance in both sexes, while adiposity and blood pressure provided limited additional explanatory value. Longitudinal and intervention studies are needed to determine whether magnesium contributes to cognitive aging independently of overall diet and whether its effects differ according to sex, nutritional status, or baseline magnesium status.

## Figures and Tables

**Table 1:** Descriptive statistics of the study sample.

Variable	Unweighted *n*	Weighted Proportion or Mean (95% CI)
Sex (M/F)	M 1153 F 1244	47% (44.4, 49.4) 53% (50.6, 55.6)
Age (years)	2397	69.1 (68.6, 69.7)
Body Mass Index (kg/m^2^)	2362	29.3 (28.8, 29.8)
Upper Leg Length (cm)	2279	37.6 (37.4, 37.8)
Upper Arm Length (cm)	2319	37.5 (37.3, 37.7)
Arm Circumference (cm)	2318	32.7 (32.3, 33.0)
Waist Circumference (cm)	2289	102.9 (101.7, 104.1)
Average Sagittal Abdominal Diameter (cm)	2224	23.7 (23.4, 24.0)
Fasting Glucose (mg/dL)	1147	112.1 (107.5, 116.6)
Magnesium Intake (mg/day)	2397	294.2 (283.3, 305.1)
Magnesium Intake from supplements (mg/day)	2397	48.3 (40.6, 56.0)
Magnesium Intake plus supplements (mg/day)	2397	342.5 (327.4, 357.6)
Systolic Blood Pressure (mmHg)	1086	131.7 (130.0, 133.5)
Diastolic Blood Pressure (mmHg)	1086	69.6 (68.3, 70.8)

Unweighted *n* represents the number of participants with available data for each variable. Continuous variables are presented as survey-weighted proportions (for sex) and means with 95% confidence intervals (95% CI), accounting for the NHANES complex sampling design. Sample size varies across variables because of missing data. CI, confidence interval; M, male; F, female.

## Data Availability

These datasets are publicly available at https://wwwn.cdc.gov/nchs/nhanes/ (accessed on 10 October 2026).

## Supplementary Materials

The following supporting information can be downloaded at https://www.mdpi.com/article/10.3390/biology15211906/s1. Supplemental Table S1. Rotated factor loadings and uniqueness values from a principal component analysis (PCA). Supplemental Table S2. Comparison of standardized coefficients from sex-stratified structural equation models with and without adjustment for energy intake. Supplemental Figure S1. Anti-Image Covariance Matrix Heatmap of partial covariances among cognitive test variables. Supplemental Figure S2. Sex-stratified structural equation models of total magnesium intake and memory performance without adjustment for energy intake.

## Abbreviations

The following abbreviations are used in this manuscript:

AA	Associate’s Degree
Aβ	Amyloid-beta
AfrAm	African American
Arm circ	Arm circumference
ATP	Adenosine triphosphate
BIC	Bayesian Information Criterion
BMI	Body Mass Index
BP	Blood Pressure
CERAD	Consortium to Establish a Registry for Alzheimer’s Disease
CFI	Comparative Fit Index
CFQ	Cognitive Functioning Questionnaire
CI	Confidence Interval
DBP	Diastolic Blood Pressure
ERB	Ethics Review Board
F1mp	Factor 1 (Memory Performance)
GED	General Educational Development
KMO	Kaiser-Meyer-Olkin (Measure of Sampling Adequacy)
MEC	Mobile Examination Center
Mg	Magnesium
MgI	Magnesium Intake
MexAm	Mexican American
MoCA	Montreal Cognitive Assessment
NCHS	National Center for Health Statistics
NHANES	National Health and Nutrition Examination Survey
NMDA	N-Methyl-D-Aspartate (Receptor)
PCF	Principal-Component Factor Analysis with Varimax Rotation
Q1, Q3	Quartiles 1 and 3 (Interquartile range)
RMSEA	Root Mean Square Error of Approximation
R^2^	Coefficient of Determination
Sag Abd	Sagittal Abdominal Diameter
SBP	Systolic Blood Pressure
SD	Standard Deviation
SEM	Structural Equation Modeling
SRMR	Standardized Root Mean Square Residual
