# Supplementary material for: Magnesium Intake, Sociodemographic Determinants, and Memory Performance in Older Adults: A Sex-Stratified Analysis in NHANES 2011–2014
Source: Biology (Basel). 2026 Oct 22;15(21):1906. doi: 10.3390/biology15211906 (PMC13762245; doi:10.3390/biology15211906)
Supplement: Supplementary file 1 [file biology-15-01906-s001.zip › biology-4577181-supplementary.pdf]

Supplemental material

Magnesium Intake, Sociodemographic Determinants, and Memory Performance in Older Adults: A Sex-Stratified Analysis in NHANES 2011–2014

Supplemental Table S1. Rotated factor loadings and uniqueness values from a principal component analysis (PCA).

Supplemental Table S2. Comparison of standardized coefficients from sex-stratified structural equation models with and without adjustment for energy intake.

Supplemental Figure S1. Anti-Image Covariance Matrix Heatmap of partial covariances among cognitive test variables.

Supplemental Figure S2. Sex-stratified structural equation models of total magnesium intake and memory performance without adjustment for energy intake.

Variable	Factor1	Factor2	Uniqueness
cfdcst1	0.8035		0.3455
cfdcst2	0.8671		0.24
cfdcst3	0.8636		0.2512
cfdcsr	0.8425		0.2706
cfdcit1		0.6965	0.5144
cfdcit2		0.7994	0.3516
cfdcit3		0.7762	0.3814
cfdcir		0.7131	0.4726
cfdast	0.58		0.6631

Supplemental Table S1. Rotated factor loadings and uniqueness values from a principal component analysis (PCA) with Varimax rotation, identifying two latent cognitive factors. Factor 1 represents verbal memory performance, with strong loadings from immediate recall trials (cfdcst1–3), delayed recall (cfdcsr), and animal fluency (cfdast). Factor 2 reflects verbal intrusion/ interference, with strong loadings from intrusion errors during immediate (cfdcit1–3) and delayed (cfdcir) recall tasks. Uniqueness of values indicate the proportion of variance in each variable not explained by the two retained factors. Variables with loadings ≥ 0.40 were considered salient contributors to the respective factor. cfdcst1–3: First, second, and third trials of CERAD word list immediate recall. cfdcsr: CERAD delayed recall. cfdcit1–3: Intrusion errors during immediate recall (trials 1–3). cfdcir: Intrusion errors during delayed recall. cfdast: Animal fluency test (total correct animals named in 60 seconds)

Supplemental Table S2. Comparison of standardized coefficients from sex-stratified structural equation models with and without adjustment for energy intake.

Outcome	Predictor	Men: No energy β (95% CI)	Men p	Men: With energy β (95% CI)	Men p	Women: No energy β (95% CI)	Women p	Women: With energy β (95% CI)	Women p
Memory performance (F1mp)	Total MgI	0.043 (−0.056, 0.142)	0.387	−0.008 (−0.141, 0.125)	0.901	0.080 (−0.018, 0.178)	0.105	0.036 (−0.070, 0.141)	0.496
Memory performance (F1rec)	Education	0.188 (0.115, 0.261)	**<0.001***	0.184 (0.109, 0.258)	**<0.001***	0.239 (0.133, 0.345)	**<0.001***	0.235 (0.135, 0.336)	**<0.001***
Memory performance (F1rec)	Household income	0.093 (−0.015, 0.201)	0.090	0.088 (−0.022, 0.199)	0.113	0.082 (−0.031, 0.195)	0.151	0.086 (−0.021, 0.193)	0.110
Memory performance (F1rec)	Age	−0.337 (−0.43, −0.24)	**<0.001***	−0.322 (−0.424, −0.220)	**<0.001***	−0.307 (−0.409, −0.206)	**<0.001***	−0.304 (−0.41, −0.20)	**<0.001***
Memory performance (F1rec)	Energy intake (per 100 kcal/day)	Not included		0.092 (−0.019, 0.203)	0.101	Not included		0.082 (−0.048, 0.213)	0.207
Total MgI	Education	0.140 (0.052, 0.227)	**0.003***	0.068 (−0.015, 0.152)	0.104	0.173 (0.086, 0.261)	**<0.001***	0.103 (0.026, 0.181)	**0.010***
Total MgI	Household income	0.124 (0.032, 0.215)	**0.010***	0.058 (−0.031, 0.148)	0.195	0.075 (−0.012, 0.161)	0.089	0.081 (0.007, 0.155)	**0.033***
Total MgI	Age	−0.022 (−0.126, 0.072)	0.633	0.075 (−0.038, 0.188)	0.184	0.022 (−0.056, 0.100)	0.566	0.036 (−0.033, 0.106)	0.297
Total MgI	Energy intake (per 100 kcal/day)	Not included		0.570 (0.508, 0.632)	**<0.001***	Not included		0.525 (0.446, 0.604)	**<0.001***

Models included total magnesium intake (MgI) from dietary and supplemental sources and were adjusted for education, household income, and age. The energy-adjusted model additionally included mean daily energy intake (per 100 kcal) as a predictor of total magnesium intake and memory performance. Analyses accounted for the NHANES complex survey design and included 1,831 adults aged ≥60 years (835 men and 996 women). Standardized coefficients (β), 95% confidence intervals (CI), and p-values are reported.
*Significant p-values.

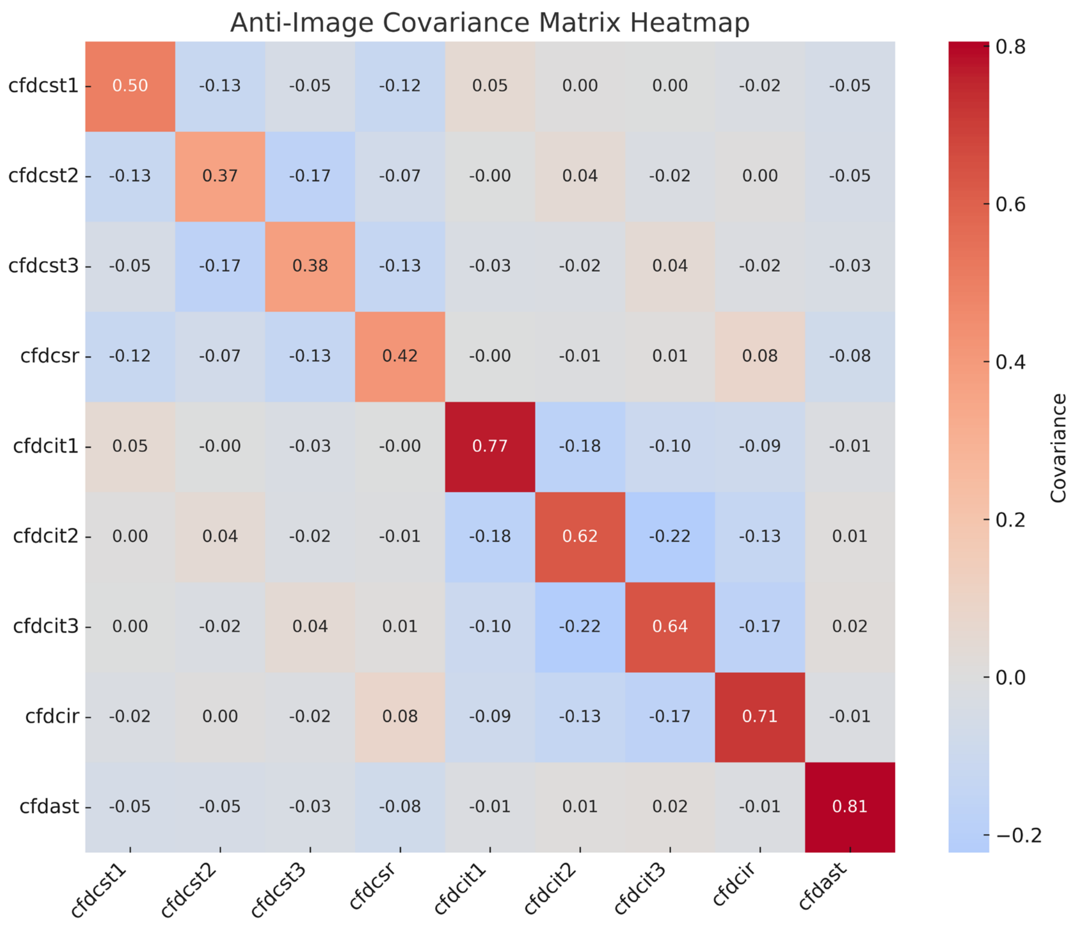

Supplemental Figure S 1 . Anti-Image Covariance Matrix Heatmap of partial covariances among cognitive test variables. The red shades represent positive partial covariances, and cooler (blue) shades indicate negative partial covariances. Diagonal elements reflect the unique variance of each variable, serving as a measure of sampling adequacy (MSA). High diagonal values suggest a variable is suitable for inclusion in factor analysis. Abbreviations: cfdcst1–3 = immediate recall trials 1–3; cfdcsr = delayed recall; cfdcit1–3, cfdcir = intrusion errors; cfdast = animal fluency task.

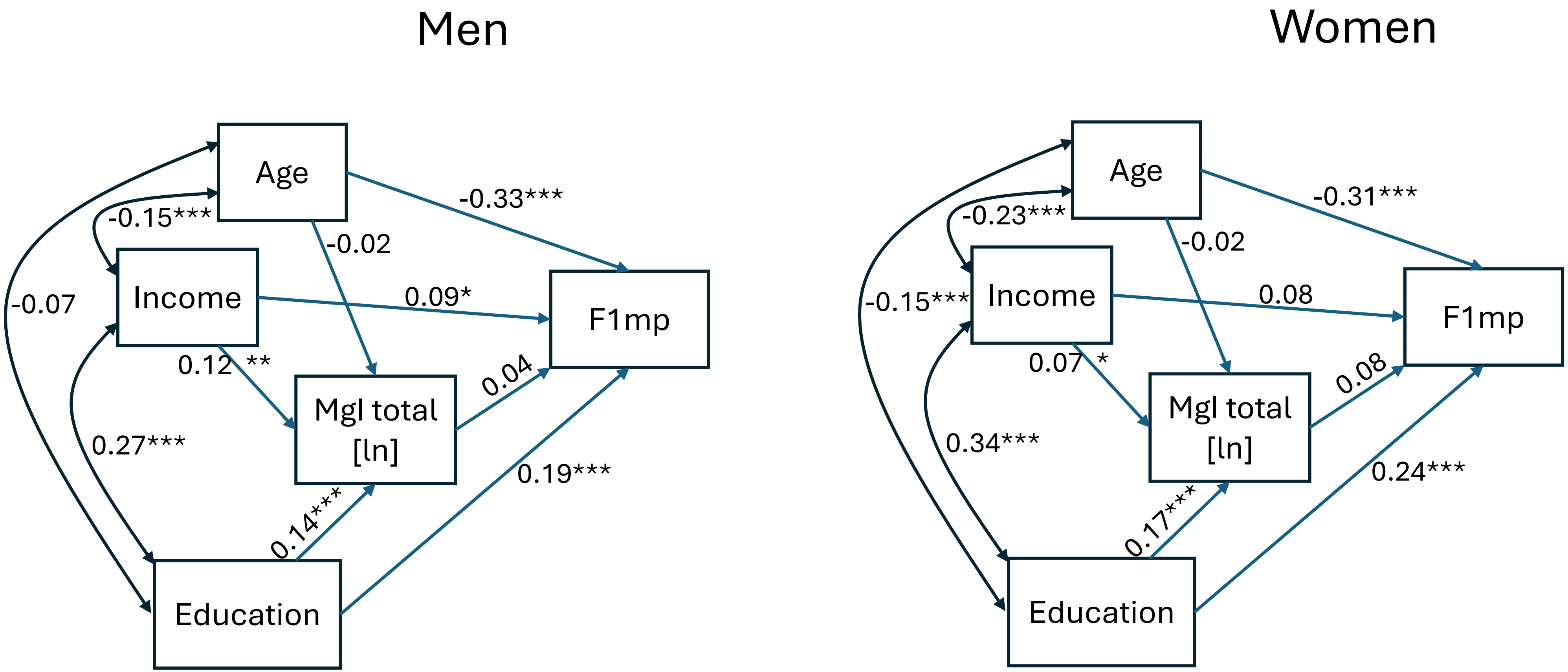

Supplemental Figure S2. Sex-stratified structural equation models of total magnesium intake and memory performance without adjustment for energy intake.

The path diagrams show the associations of education, household income, and age with total magnesium intake (dietary plus supplemental sources) and memory performance (F1mp) among men and women. Total magnesium intake was log-transformed. Values along the paths represent standardized coefficients (β). Curved double-headed arrows represent covariances among sociodemographic variables. Analyses accounted for the NHANES complex survey design. *$p < 0.10$, **$p < 0.05$, and ***$p < 0.001$
